# Application of body area network wearable smart bracelet in epidemic isolation scenario

**DOI:** 10.2478/jtim-2024-0010

**Published:** 2024-07-27

**Authors:** Wei Han, Hongbin Cai, Jiafa Lu, Le Yang, Yu Pang, Jiaqin Fang, Haojun Fan, Shike Hou

**Affiliations:** Institute of Disaster and Emergency Medicine, Tianjin University, Tianjin, 300072, China; Emergency Department of Shenzhen University General Hospital, Shenzhen, Guangdong, 518055, China; Chongqing University of Posts and Telecommunications, Chongqing, 400065, China; School of Microelectronics, South China University of Technology, Guangzhou, Guangdong, 510641, China

## To the editor

In the context of Corona Virus Disease 2019 (COVID-19), it is challenging to continuously and effectively monitor the body temperature and position of key isolated personnel while reducing the contact between medical staff and patients. This paper proposes a new technical scheme for abnormal personnel monitoring such as vital signs combined with 5th Generation Mobile Communication (5G), body area network and cloud technology. This scheme uses the characteristics of 5G technology, such as high rate, low delay and large connection, to realize real-time data transmission.^[1]^ Through the body area network technology, the body temperature and position information of patients can be monitored in real time without touching them.^[2]^ At the same time, the use of cloud technology data storage and processing advantages, greatly reduce the workload of managers. In order to achieve this solution, the team developed a smart bracelet based on 5G and body area network. The bracelet has the characteristics of portability, automation and digitalization, which can effectively reduce the contact between medical staff and patients and reduce the risk of nosocomial infection.

Forty samples of suspected cases, high-risk patients and caregivers of patients in isolation wards admitted to the Emergency department of Shenzhen University General Hospital during the outbreak of COVID-19 in 2020, as shown in Table 1. The subjects were divided into axillary temperature group and bracelet group, camera group and bracelet group for comparison. Ethical approval and subject consent were obtained for the study.

**Table 1:** Basic data of the study subjects

Age	51.7 ± 18.8
Proportion of males (%)	62.5
High risk cases of COVID-19 (%)	77.5
Diagnosed respiratory diagnosed (%)	12.5
Diagnosed digestive system diseases (%)	22.5
Diagnosed infection in other sites (%)	22.5
Escort personnel (%)	22.5

*P* < 0.05.

**Figure 1:**
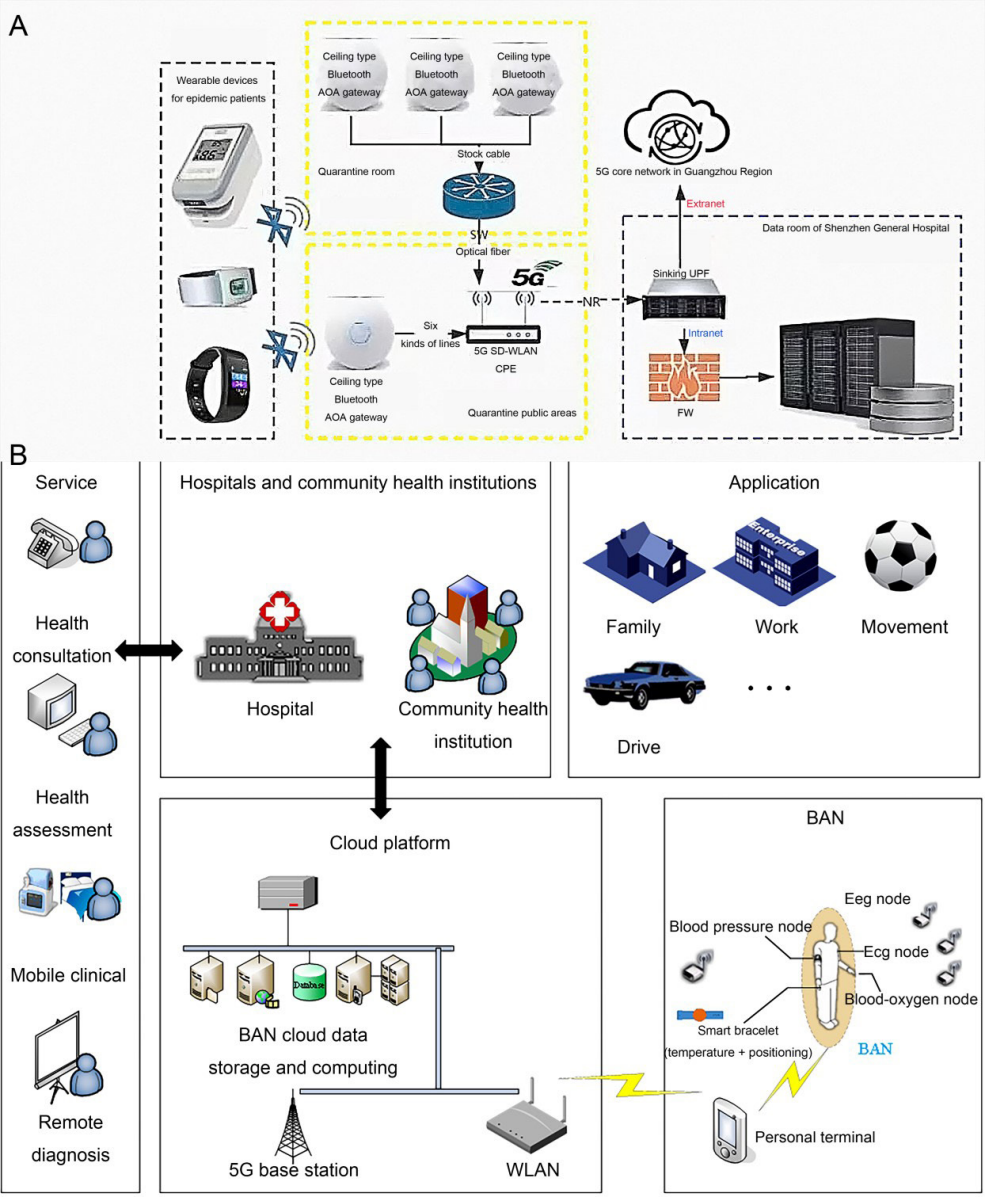
5G and Bluetooth AOA positioning scheme networking diagram (A) and BAN-based remote health monitoring system architecture (B) 5G, 5th Generation Mobile Communication; AOA, Angle of Arrival; BAN, The body area network.

The design of the smart bracelet includes a body temperature sensor and a positioning chip. The map shows the warning area centered on the quarantine area, and the warning is triggered when the boundary is crossed. Body temperature and position data were collected at a fixed frequency every 5 min, and abnormal body temperature and position were reported by the system. 5G+ body area network technology scheme, Figure 1 (A) uses multiple indoor Bluetooth Angle of Arrival (AOA) gateways to achieve accurate positioning of small range of people, and is suitable for big data transmission combined with 5G. Figure 1 (B) Vital signs parameters are collected by wearable devices and sent to the cloud platform for processing through communication networks such as the body area network (BAN) and 5G to realize remote diagnosis and other services. In the cloud technology scheme, the body temperature and location of the isolated personnel were collected in real time through wearable devices, and the information was stored and transmitted in real time in the cloud. The system can cover isolation hospitals, community isolation buildings and factory dormitories, *etc*., with electronic fence function and drop detection, and timely notification of relevant personnel.

For temperature measurement, the axillary temperature of the study subjects was manually collected by nursing staff using a Shanghai brand mercury thermometer at fixed time points (7: 30 am and 2: 00 PM) daily. Before temperature measurement, ensure that the patient is in a resting state to avoid interfering factors. The recorded data were divided into axillary and bracelet temperature groups. Position measurement, the smart bracelet prompts the video when outside the warning range, and the camera monitors the bracelet displacement system when the patient leaves the monitoring area. Patients were negative within the monitored area and positive otherwise. The method is divided into two groups: camera and bracelet monitoring. SPSS 26.0 software was used for data analysis. Intraclass Correlation Efficient (ICC) was used to evaluate the consistency of temperature test data between the two groups, and Kappa test was used to compare the consistency of position results between the two groups. The sensitivity, specificity, predictive value and likelihood ratio were calculated to evaluate the positioning accuracy of the device. Differences were considered significant at Analysis of body temperature monitoring results of smart bracelet. In this study, there were 31 suspected cases (77.5%) and 9 accompanying persons (22.5%). A total of 226 person-times of body temperature were monitored by smart bracelet and mercury thermometer, of which 20person-times of body temperature monitoring by mercurythermometer ≥ 37.3 ℃accounted for 8.5%. There were14 times of body temperature monitoring values ≥ 37.3℃,accounting for 6.2%. According to the intraclass correlation coefficient (ICC), the ICC of the axillary temperature group and the bracelet temperature group was 0.883, indicating that the two temperature measurement methods had high consistency and accuracy (*P* < 0.05). Analysis of the positioning results of the smart bracelet. The system reported a total of 790 person-times of bracelet positioning. The negative display of the bracelet was 767, and the negative report of the camera monitoring group was 762. The consistency test showed that the results of the bracelet report and the results of the camera monitoring group were consistent, and the Kappa value was 0.494, and the consistency of the two methods was medium. The sensitivity was 46.4%, the specificity was 98.7%, the positive predictive value was 0.565, the negative predictive value was 0.980, the positive likelihood ratio was 35.692, the negative likelihood ratio was 0.530, and the accuracy was 0.968. The bracelet positioning technology had a better performance in accuracy.

Annie Anak Joseph and Swamy *et al*.^[3,4]^ showed that the wristband body temperature monitoring system is reliable in practice. This study compared the measurement results of mercury thermometer and bracelet in 40 subjects, and found that the intraclass correlation coefficient ICC was 0.883, indicating that the two methods were consistent and accurate. Chao Sun *et al*.^[5]^ proposed a 5G-GNSS hybrid positioning scheme based on AOA-TOA measurement, which is not affected by time synchronization errors and improves the accuracy and robustness of the hybrid positioning system. In this study, the wristband indoor AOA positioning and outdoor Beidou positioning technology were used to monitor the range of motion of the isolated patients. The Kappa value between the wristband positioning results and the results reported by the camera monitoring group was 0.494, the specificity was 98.7%, and the accuracy was 0.968. The accuracy of bracelet positioning technology is high, but the consistency needs to be improved. In the future, the positioning technology of the bracelet can be optimized to improve the consistency and accuracy, so as to better support isolation measures and epidemic prevention and control.

The smart bracelet can remotely monitor the body temperature and position of large-scale patients without physical contact. However, it still has some limitations. The bracelet’s temperature measurement may be lower than that of a mercury thermometer, possibly due to room temperature and wearing issues. The positioning error is related to system ambiguity and subject activity. The direction of improvement includes improving sensor accuracy, adopting advanced positioning techniques, and optimizing algorithms to reduce interference. Higher device costs, potential 5G network limitations, battery life concerns, and so on are factors to consider. The optimization plan involves enhancing sensor accuracy and positioning algorithms, reducing equipment manufacturing costs, optimizing 5G network coverage and readiness for alternative networks, and employing low-power design and fast charging technology. Although the device does not confirm the diagnosis, it is valuable in the study of symptoms such as fever, especially in susceptible diseases.^[6, 7, 8]^ Bluetooth AOA indoor positioning technology reduces risk, manpower, and medical costs. 5G increases transmission rates and supports real-time analysis. With the assistance of BAN technology, it is possible to monitor the real-time body temperature and location information of individuals without the need for direct contact. Simultaneously, the outstanding data storage and processing capabilities of cloud technology significantly alleviate the workload of management personnel.^[9]^ Although designed for COVID-19, it is also applicable to other infectious diseases. The equipment is widely used in hospitals, schools, factories, *etc*., which provides strong support for epidemic prevention and control.

In this study, the intelligent bracelet designed by 5G+ body area network can accurately monitor the body temperature and displacement of key people in the emergency ward, which can replace some of the work of medical staff. At the same time, it can also play a key role in the prevention and control of other serious infectious diseases and emerging infectious diseases. By adding functional modules such as pulse monitoring, oxygen saturation and blood pressure, the device can be widely used in more medical fields to improve the diagnosis and treatment effect.
